# Boosting Thermoelectric Performance in Nanocrystalline Ternary Skutterudite Thin Films through Metallic CoTe_2_ Integration

**DOI:** 10.1021/acsami.3c17695

**Published:** 2024-03-15

**Authors:** Bhawna Jarwal, Suman Abbas, Ta-Lei Chou, Suneesh M. Vailyaveettil, Ashutosh Kumar, Shaham Quadir, Thi-Thong Ho, Deniz P. Wong, Li-Chyong Chen, Kuei-Hsien Chen

**Affiliations:** † Molecular Science and Technology Program, Taiwan International Graduate Program, Academia Sinica, Taipei 10617, Taiwan; ‡ International Graduate Program of Molecular Science and Technology, National Taiwan University, Taipei 10617, Taiwan; § Institute of Atomic and Molecular Sciences, Academia Sinica, Taipei 10617, Taiwan; ∥ Center for Condensed Matter Sciences, National Taiwan University, Taipei 10617, Taiwan; ⊥ Department of Physics, National Central University, Taoyuan 32001, Taiwan; # Department of Materials Science and Metallurgical Engineering, Indian Institute of Technology Bhilai, Durg, Chhattisgarh 491001, India; ∇ Materials Science Center, National Renewable Energy Laboratory (NREL), Golden , Colorado 80401, United States; ○ Helmholtz-Zentrum Berlin für Materialien und Energie, Hahn-Meitner-Platz 1, Berlin D-14109, Germany; ◆ Department of Physics, National Taiwan University, Taipei 10617, Taiwan; ¶ Center of Atomic Initiative for New Materials, National Taiwan University, Taipei 10617, Taiwan

**Keywords:** ternary skutterudite, binary telluride, metallic phase, nanocomposite, work function, band bending, interface scattering

## Abstract

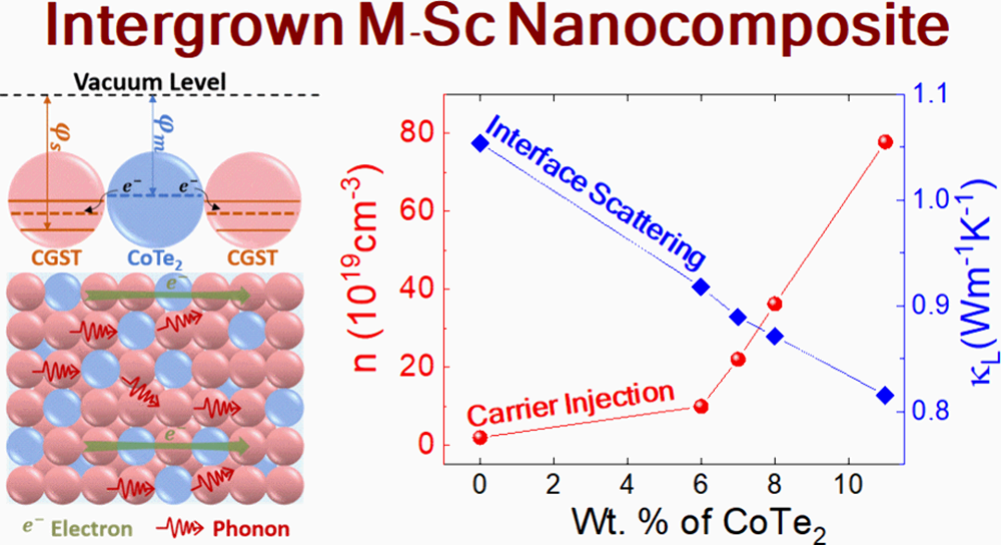

Metal–semiconductor nanocomposites have emerged as a viable strategy for concurrently tailoring both thermal and electronic transport properties of established thermoelectric materials, ultimately achieving synergistic performance. In this investigation, a series of nanocomposite thin films were synthesized, embedding metallic cobalt telluride (CoTe_2_) nanophase within the nanocrystalline ternary skutterudite (Co(Ge_1.22_Sb_0.22_)Te_1.58_ or CGST) matrix. Our approach harnessed composition fluctuation-induced phase separation and in situ growth during thermal annealing to seamlessly integrate the metallic phase. The distinctive band structures of both materials have developed an ohmic-type contact characteristic at the interface, which raised carrier density considerably yet negligibly affected the mobility counterpart, leading to a substantial improvement in electrical conductivity. The intricate balance in transport properties is further influenced by the metallic CoTe_2_ phase’s role in diminishing lattice thermal conductivity. The presence of the metallic phase instigates enhanced phonon scattering at the interface boundaries. Consequently, a 2-fold enhancement in the thermoelectric figure of merit (zT ∼ 1.30) is attained with CGST-7 wt. % CoTe_2_ nanocomposite film at 655 K compared to that of pristine CGST.

## Introduction

Harnessing waste heat from traditional energy sources represents a significant opportunity for the advancement of eco-friendly energy generation. Thermoelectric technology has gained attention for its ability to convert waste heat into electricity efficiently, supporting clean renewable energy production. The efficiency of a thermoelectric (TE) material is directly related to the dimensionless figure of merit,  (where *S* is the Seebeck coefficient, σ is the electrical conductivity, *T* is the absolute temperature, and κ_total_ is the total thermal conductivity; κ_total_ = κ_L_ + κ_e_, κ_L_, and κ_e_ are the lattice and electronic part of thermal conductivity).^1−4^ However, achieving a favorable zT remains a difficult task due to the interplay among these transport properties, which tend to counterbalance each other. One effective strategy for decoupling these properties involves the manipulation of nanoscale crystallinity and integration of nanostructures into the parent material. This results in the creation of distinctive multiphase structures designed to modify carrier transport.^5−8^ The presence of a multiphase interface disrupts the heat-carrying phonon movement due to varying thermal transport properties across different lattices, especially at the nanoscale where electron and phonon mean free paths differ significantly. A nanoscale phase with a coherent interface is essential for inducing long-wavelength phonon scattering while preserving the mobility of charge carriers.^3,9^ The application of such multiphase nanocomposites facilitates synchronized modulation of electron and phonon transport by establishing unique heterojunctions at the interface between the parent matrix and the guest phase.^7^

Building upon these advancements, many recent studies have explored thin film synthesis to further exploit the effects of interfaces and grain boundaries in low-dimensional systems to strengthen the decoupling effect on the TE properties. Besides, thin film-based TE microgenerators are increasingly gaining prominence, particularly for potential IoT applications such as sensors, micropower sources, and flexible devices providing an ideal application domain for thin film TE devices. These devices are envisioned to offer sustainable, self-powered, and maintenance-free solutions for powering miniaturized machines and accessories.^10−12^ A variety of thermoelectric (TE) materials have been developed into thin films, reflecting modern material science’s focus on effective and sustainable solutions. Notably, bismuth telluride-based thin films have shown promising results, with high thermoelectric figures of merit (ZTs) above 1 for n-type and 1.5 for p-type at temperatures below 500 K.^13−15^ However, their optimal performance in lower temperature regions typically near room temperature underscores the ongoing challenges in TE material development for midtemperature applications. This highlights the trade-offs in optimizing for specific conditions and the continued effort to develop TE materials suitable for a broader range of applications.

CoSb_3_-based skutterudites have emerged as a promising TE candidate, especially for intermediate-temperature applications due to their tunable properties in bulk^16−18^ and thin films.^19−23^ Various strategies such as doping,^24−27^ void filling,^28−31^ and isoelectronic anion substitution^32−35^ have been explored to enhance their TE properties. The isoelectronic anion substitution involves replacing Sb atoms with elements from groups IV and VI, causing a distortion in the Sb rings and altering the crystal lattice from cubic *Im*3̅ to rhombohedral *R*3̅ symmetry. This distortion causes significant reduction in lattice thermal conductivity, also harms electronic conduction. In the pursuit of improved electronic properties, researchers have investigated ternary skutterudite with extrinsic doping.^36−39^ While substantial progress has been achieved in improving the thermoelectric (TE) properties of ternary skutterudite-based materials in their bulk form, a lack of reports on the ternary skutterudite-based thin films underscores a promising research avenue.

However, thermoelectric performance of thin film counterpart does not align with their bulk form due to different fabrication process lead to dissimilar electronic and thermal transport properties.^19^ To enhance the thermoelectric properties of thin films, developing metal–semiconductor (M–Sc)-based composites offers a viable approach, extensively researched in the bulk forms of various thermoelectric materials.^40−45^ These composites effectively boost the electrical conduction through mechanisms such as carrier channeling, and carrier injection simultaneously suppresses phonon conduction by enhancing interfacial phonon scattering to achieve enhanced thermoelectric performance.^9,46,47^ The influence of the metallic phase on σ is contingent upon the band structure of the matrix, the potential energy barrier at the interface between the two phases, and the electrical and thermal transport properties of the metallic phase. Also, the morphology, amount, and physical characteristics of the guest phase play a critical role in determining the resulting thermoelectric transport properties.^9,48−51^ Previous studies had demonstrated the effect of metallic nanostructures in TE materials like (Bi,Sb)_2_(Te,Se)_3_,^42^ CoSb_3_,^43^ and MnSi_1.787_Al_0.0138_,^44^ where the selection of metallic inclusions with distinct work functions provides the perspective of a tunable barrier at the interface, promoting the electronic transport to tailor the TE properties. Consequently, the investigation of multiphase composite structures emerges as one of the most viable strategies to enhance the thermoelectric performance of existing materials.

Herein, we developed an optimized ternary skutterudite Co(Ge_1.22_Sb_0.22_)Te_1.58_ (CGST) with Sb doping as the pristine phase and their nanocomposite thin films along with in situ formed and uniformly distributed metallic CoTe_2_. We had employed co-sputtering as an efficient time-saving fabrication method followed by annealing treatment to synthesize nanocrystalline thin films. To enhance σ of the nanocrystalline ternary skutterudite thin film, incorporating metallic CoTe_2_ with a higher carrier density presents a suitable approach due to the restricted carrier mobility of the nanocrystalline matrix. Our findings indicate that incorporating CoTe_2_ into the composite simultaneously enhances both electrical and thermal properties. This improvement is associated with the formation of a unique microstructure through crystallization, a process driven by composition fluctuation-induced phase separation and subsequent growth during thermal annealing, a novel observation in the context of skutterudite-based thin films. The analyzed results demonstrate an optimized interface potential barrier with an ohmic-type characteristic at the CGST and CoTe_2_ interface, enabling charge spillover, increasing effective carrier density of the nanocomposites. Additionally, the coherent coexistence of nano-CoTe_2_ grains alongside CGST grains had shown a minimal influence on mobile charge carriers. Besides, the interface boundary significantly contributes to phonon scattering, leading to reduction in lattice thermal conductivity. Hence, a zT value of 1.30 at 655 K has been achieved for the multiphase composite system consisting of a semiconducting CGST matrix phase and a metallic CoTe_2_ phase. This work highlights the potential of nanocomposite structures with uniformly distributed nanostructures as a feasible and promising strategy for enhancing thermoelectric performance and bringing us closer to achieve efficient and sustainable thermoelectric energy conversion systems.

## Experimental Section

A series of Co(Ge_1.22_Sb_0.22_)Te_1.58_ or CGST with varying weight percent of CoTe_2_ nanocomposite thin films had been studied in this work, named as *x* = 0, 6, 7, 8, and 11, where “*x*” represents the weight percentage of CoTe_2_. The thin films were prepared by using a physical vapor deposition method, co-sputtering. Pure Co and alloy Ge_19_Sb_2_Te_22_ targets were used to co-sputter on c-plane sapphire substrate with a tunable power ratio to attain optimized conditions. The as-deposited samples were further crystallized by post annealing treatment at 773 K for 1 h. The phase formation and their respective crystal structure of composite thin films were studied by high-resolution synchrotron X-ray diffraction measurement carried out at TPS 19A beamline of National Synchrotron Radiation Research Center (NSRRC), Taiwan. The diffraction pattern of the thin film samples was collected on a nine-circle diffractometer at 20 keV (λ = 0.61992 Å) source energy using a multicrystal analyzer detector mounted on a goniometer head, over a 2θ range of 5–25° with a step size of ∼0.008°. The collected diffraction patterns were refined by full pattern Rietveld refinement using Topas V5.0 software to estimate the respective phase content.

The morphology and microstructure were determined by a field emission scanning electron microscope (FESEM, JEOL 6700F). The composition data were collected using a field emission electron probe microanalyzer, EPMA (JEOL JXA-8200). X-ray photoemission spectroscopy (XPS) and ultraviolet photoemission spectroscopy (UPS) analyses were performed on a PerkinElmer Physical Electronics (PHI 5400 spectrometer) with monochromatic Al Kα as the exciting source and equipped with a helium (*h*ν = 21.2 eV) discharge lamp. UPS has been employed to determine the work function of CGST and CoTe_2_ pristine structures to study the band alignment and interface kinetics between them. The energy scale was calibrated by setting the Fermi edge of the gold at 0 eV.

The nanocomposite interface examined under scanning transmission electron microscopy (STEM) was performed on spherical aberration-corrected JEOL 2100FX with an accelerating voltage of 200 kV and high-angle annular dark-field (HAADF) collection inner/outer semiangles of 70/190 mrad, featuring a spatial resolution of ∼0.9 Å. All HAADF images (acquisition time ∼8 s) recorded were Bragg filtered to reduce noise arising from dechanneling as a result of thickness and crystallinity. TEM samples were prepared by first scratching the powder from the thin film, which was then dissolved in methanol. This mixture underwent ultrasonication for 45 min. Subsequently, a few drops of the solution were placed onto a TEM grid for analysis. For cross-section TEM-EDS mapping, the sample was cut into a thin slice using a focused ion beam (FIB). However, the high-energy ion damage was severe, which limited further examination.

To understand the electrical properties and conduction pathway of nanocomposite films at the microscopic scale, the current map was obtained by conductive atomic force microscopy (CAFM) mode on Bruker Dimension Icon, PeakForce TUNA mode with a bias unit with the maximum current range of 500 nA. The CAFM works in contact, constant force, and constant bias mode with a scanning resolution of 1 nm. The results were obtained under 1 mV constant bias for 1 × 1 and 5 × 5 μm^2^ area. The room temperature hall coefficient (*R*_H_) was measured using a four probe assembly based on Van der Pauw method at Ecopia-HMS-3000 with a magnetic field of 0.55 T. The carrier density (*n*_H_) and hall mobility (μ_H_) values were further calculated using the formulas  and μ_H_ = σ*R*_H_, respectively. Thermoelectric properties like electrical conductivity and Seebeck coefficient were simultaneously measured by an ULVAC-RIKO ZEM-3 system under a helium atmosphere from room temperature (RT) to 673 K. The thermal conductivity data was determined in the same temperature range by using a three-layer model fitting of thermoreflectance data measured using Linseis TF-LFA. Bulk specific heat and density values were used to fit the measured data following the differential heat transport model through a multiple-layer system. The estimated measurement uncertainties were 6% for the Seebeck coefficient, 8% for the electrical resistivity, and 11% for the thermal conductivity.^52^

## Results and Discussion

**Figure 1:**
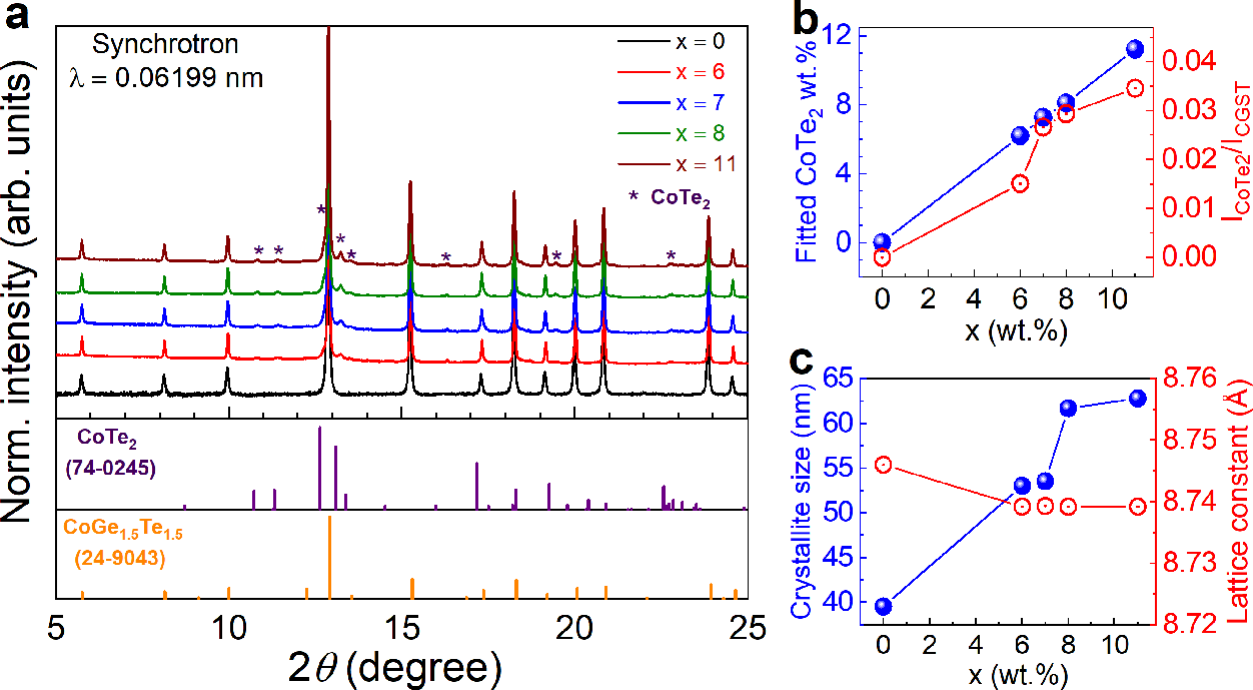
(a) SP-XRD patterns of CGST with *x* wt. % of CoTe_2_ (*x* = 0, 6, 7, 8, and 11) composites at RT, (b) weight fraction of CoTe_2_ calculated by Rietveld refinement and intensity ratio of CoTe_2_ (120)/ CGST (311) peak, and (c) composition dependent lattice parameter and crystallite size using Scherrer’s equation.

The optimized pristine ternary skutterudite CGST thin film and metallic CoTe_2_-embedded nanocomposite thin films with varying weight percentages were synthesized. The present study demonstrates that doping the ternary skutterudite with Sb effectively restores its original cubic structure from the distorted symmetry, which aligns with previous findings.^36^ This structural change was investigated using synchrotron X-ray diffraction and Cs-corrected scanning transmission electron microscopy-high-angle annular dark-field (STEM-HAADF) imaging techniques. The HAADF images of the CGST phase along [111] and [100] crystallographic directions, are highlighted in Figure S1a,b. These images showcase a well-crystallized CGST lattice resembling the CoSb_3_ framework.^23^ Furthermore, the phase structures of the pristine CGST and nanocomposite films are represented in Figure 1a. The diffraction peaks of all samples can be well indexed to the near ideal cubic (*Im*3̅) lattice, as no reflection planes from *R*3̅ symmetry have been observed. In addition to the skutterudite phase, diffraction peaks of CoTe_2_ (orthorhombic, *Pnnm*) were observed in *x* = 6, 7, 8, and 11. The relative percentage of the CoTe_2_ phase in all composite films was estimated by Rietveld refinement using TOPAS V5 software. The respective refinement pattern and resulting difference curves are represented in Figure S2 and summarized in Table S1 providing the lattice constants and *R*_wp_ values corresponding to the best-fit model. The composition results obtained from EPMA of the annealed nanocomposite thin films show an increased off-stoichiometry of elemental distribution as the cosputtering power ratio tuned away from pristine film conditions (Table S2). This off-stoichiometry promotes the phase separation between the abovementioned two phases, which suggests a narrow composition zone for the pure CGST phase and encourages the formation of the cobalt telluride.^53^ This observation has been confirmed by the increasing relative intensity of CoTe_2_ reflection peaks in XRD with an increase in off-stoichiometry for *x* = 6, 7, 8, and 11. Notably, the increment in relative intensity ratio of CoTe_2_ (120)/CGST (311) peaks with higher “*x*” indicates increased proportion of CoTe_2_ in nanocomposite films (Figure 1b). A similar observation has also been made by Raman analysis where no CoTe_2_ vibrational mode appeared for *x* = 0 but show CoTe_2_ Raman mode for *x* = 6, 7, 8, and 11 nanocomposite films, as shown in Figure S3.

Above and beyond, no obvious peak shift was observed for diffraction peaks of the matrix structure of all films, as shown in enlarged XRD patterns at the 2θ range between 12.7–13.0° and 23.6–24.8° in Figure S4b,c. The calculated lattice parameter of the CGST phase considering an ideal cubic lattice represented in Figure 1c shows a negligible change with respect to increasing off-stoichiometry. This insignificant difference implies that the compositional change drives only phase separation rather than affecting the pristine phase composition. Our conjecture from XRD data was well corroborated by the XPS results. The Co 2p and Te 3d core levels of the CGST nanocomposite thin films for *x* = 0 and 7 are shown in Figure S5. The 2p Co and 3d Te peaks of *x* = 7 nanocomposite film did not show any prominent shift compared to those of the pristine film, suggesting that the CoTe_2_ phase remains a separated physical entity without affecting the chemical bonding of the matrix. Furthermore, the crystallite size for all thin films was estimated using Scherrer’s Equation () to observe the effect of phase separation on crystallization. The crystallite size for nanocomposite films (Figure 1c) slightly increased between 40 to 60 nm for *x* = 0 to 11. Since all films are in the nanocrystalline region, this small change is inconsequential. Additionally, cross-sectional SEM images of all composite films are presented in Figure S6. These images reveal nanosized spherical grains comprising a compacted film with a thickness close to 400 nm, uniformly deposited on the *c*-plane sapphire substrate. Also, a cross-sectional TEM-EDS map for *x* = 7 had been provided in Figure S7, representing elemental distribution in the nanocomposite thin film.

Conductive atomic force microscopy (CAFM) provides a comprehensive analysis concerning the microstructure and electronic conduction characteristics of the composite structure. A thorough understanding of conducting networks in metal–insulator composite had been given by probing the conductance distribution of two different phases at the microscopic level.^40,54,55^ A similar study had also been applied to study the current work. During the CAFM analysis, the atomic force microscopy (AFM) tip scans the surface of the metal–semiconductor composite. The tip makes contact either with the metallic or semiconducting phase. In the case of contact with a particle, which is part of the conductive network extending to the surface, the application of a bias allows for electron flow between the sample and the tip. Consequently, a measurable current response can be collected based on the electronic conduction of each phase.^38^ This current response directly correlates with the electronic conduction of each phase. Consequently, the different values of current response in a CAFM local mapping can help to distinguish the specificity of each phase.

**Figure 2:**
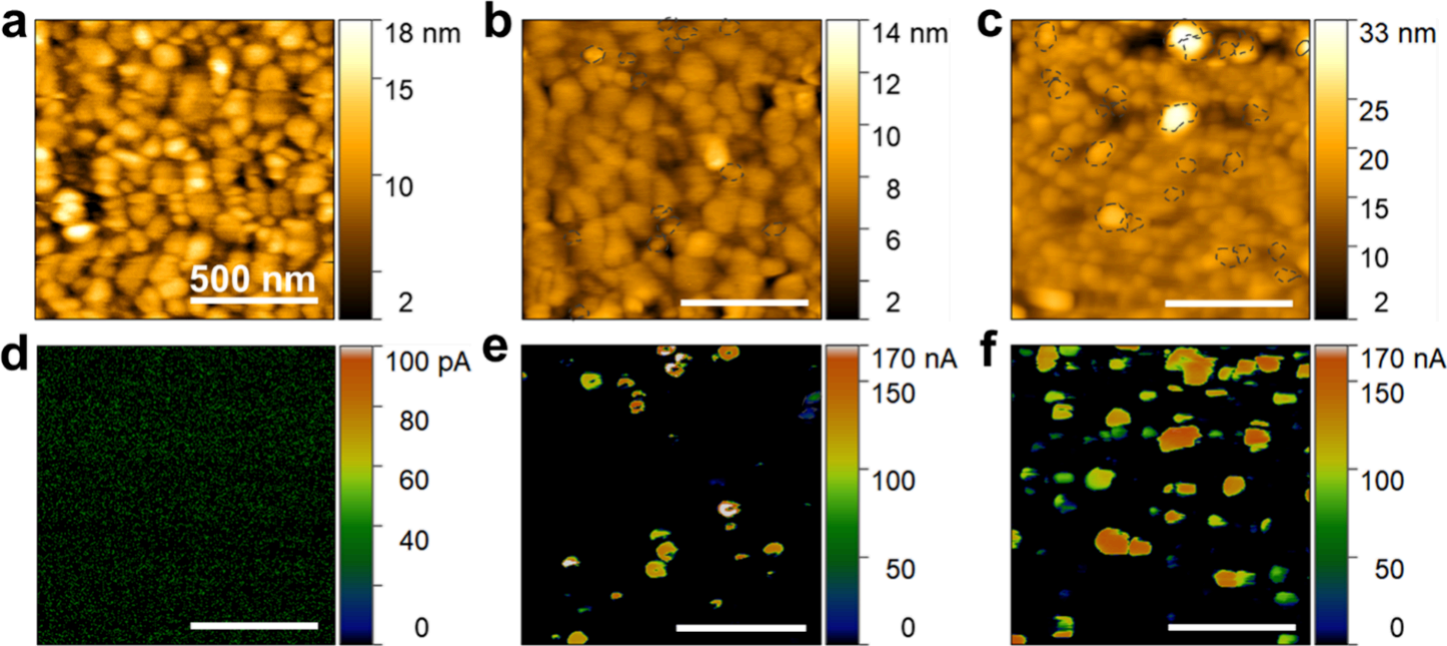
(a–c) Surface topology and (d–f) the corresponding current distribution map for nanocomposite films *x* = 0, 7, and 11.

The surface topology and corresponding current distribution map for *x* = 0, 7, and 11 thin films are shown in Figure 2a–c. All of the experimental results presented here were obtained with the same tip to avoid instrumental errors. Regardless of the phase separation, the topography shows densely packed and uniformly sized spherical nanograins in all films. The current response of pristine CGST (*x* = 0) observed under CAFM measurement was notably low, adversely affecting the signal quality and contrast in the resulting CAFM image, visible from the predominant black appearance in Figure 2d. Additionally, the green scattered points within the image are indicative of a uniform current distribution across the CGST grains. These points, while subtle, are crucial, as they highlight areas where the current response is in line with the expected behavior of CGST grains, thereby representing a uniform and consistent current distribution throughout the area under investigation. In contrast, the nanocomposite films containing metallic CoTe_2_ (*x* = 7 and 11) present nonuniform current distribution maps, with some grains exhibiting significantly higher current response (marked with dashed edges) than the surrounding grain matrix (Figure 2e,f). The current range of conducting grains falls between 100 and 150 nA for both nanocomposite films (*x* = 7 and 11) whereas the counterpart CGST grains were in 40–60 pA current range. The variation in current distribution can be attributed to the distinct electronic conduction properties of the two materials. CGST is a chalcogenide semiconductor material known for its low electrical conductivity,^36−38^ whereas CoTe_2_ is a highly conductive metallic alloy,^56^ as depicted in Figure S8b. In the composite films, the conducting grains are identified as CoTe_2_ grains, which exhibit a stronger current response compared to the CGST grains, owing to their superior conductivity. These conducting grains are uniformly distributed as shown in Figure S8 (larger investigated area under CAFM measurement) and appear to be intergrown with the CGST grains, indicating good adhesion, which can be visible in surface topography images. Additionally, the nature of these grains suggests that the CoTe_2_ grains crystallized alongside CGST grains probably form a coherent heterostructure. To explore the interface characteristic between intergrown CGST and CoTe_2_, *x* = 7 nanocomposite film was subjected to detailed analysis by high-angle annular dark-field (HAADF) imaging, with the findings illustrated in Figure S1c,d. The HAADF images reveal the presence of regions exhibiting composition-induced phase separation, leading to the nucleation and growth of distinct phases, at the microscopic level. This observation is crucial as it indicates a high degree of coherence between the CGST and CoTe_2_ phases.^57,58^ The result shows a clean interface between both phases adjacent grains, suggesting that the nanocomposite films containing intergrown nanograins of CGST and CoTe_2_ create unique microstructural inhomogeneity with smooth interface, which facilitate less destruction to charge carriers.

**Figure 3:**
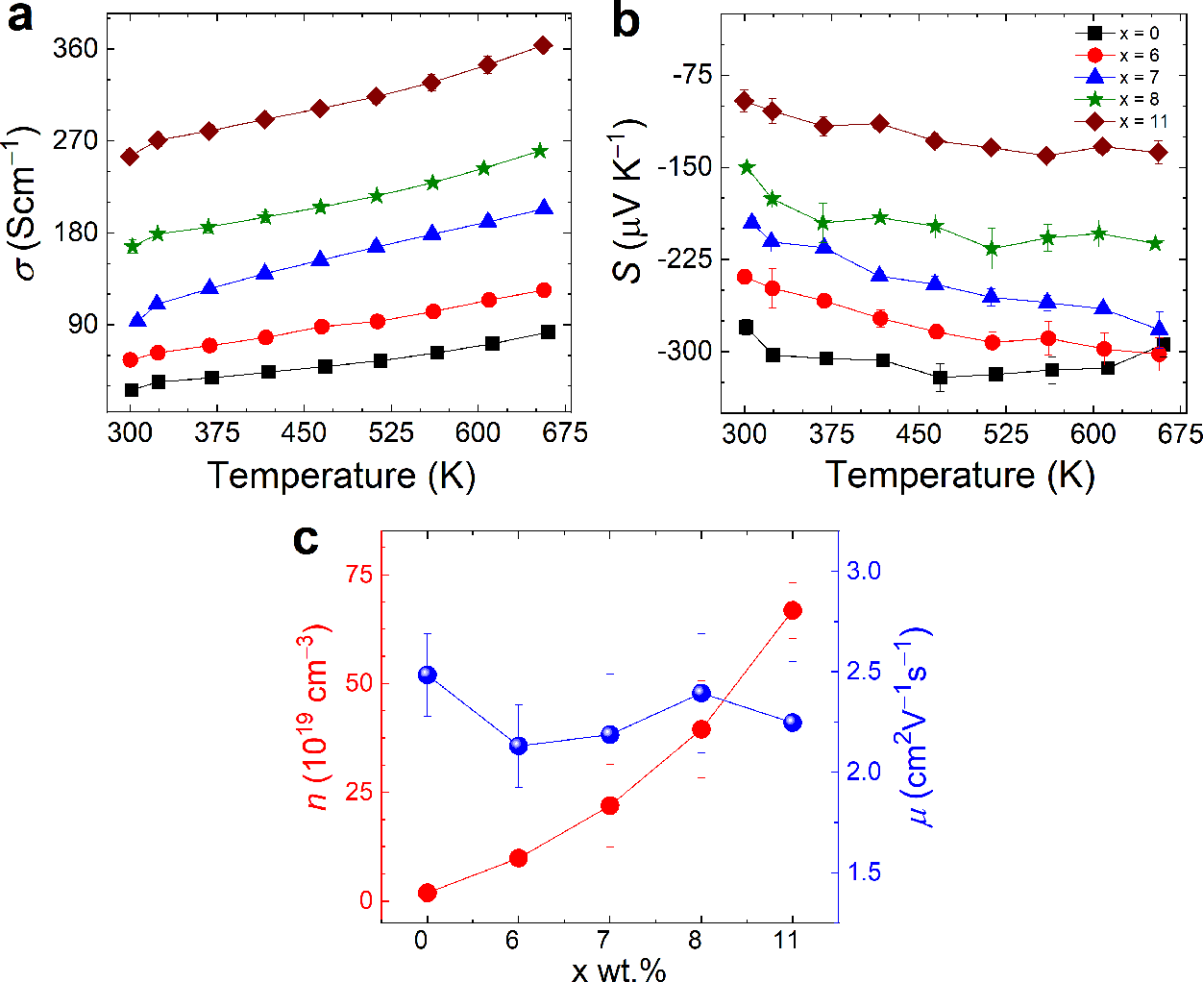
Temperature dependence of (a) electrical conductivity, (b) Seebeck coefficient, and (c) room-temperature electronic transport properties of the composite films with different “*x*”.

The aim of forming the metallic CoTe_2_ phase embedded within the semiconductor CGST matrix was to enhance the electrical conduction by manipulating the charge carrier density of the parent material through charge spillover from metallic domains having a relatively lower work function, discussed in detail in the next section. The CGST-CoTe_2_ nanocomposite systematically displayed higher electrical conductivities and lower absolute values of the Seebeck coefficient than the pristine CGST (Figure 3a,b). The observed enhancement in electrical conductivity can be attributed to the incorporation of the conducting phase CoTe_2_. The absolute σ value of the pristine CGST film is 26 S cm^–1^ (300 K), which increases to 255 S cm^–1^ for the *x* = 11 nanocomposite film. To comprehend this enhancement in electrical conductivity, the effective carrier density and Hall mobilities of all films were quantified, as illustrated in Figure 3c. The effective carrier density of the nanocomposite films displayed an upward trend with a progressive increase in the weight percentage of the binary telluride phase. This increase in carrier density is associated with the injection of free electrons from the CoTe_2_ grains. However, this increasing carrier density causes no severe impact on mobility, resulting in a negligible reduction in mobility values. This anomalous observation can be related to the presence of highly conductive CoTe_2_ grains adjacent to CGST grains facilitating charge carrier’s movement with less hindrance. These coalesced mixed phase grains with minimal distortion at their interface result in minute interfacial scattering of mobile electrons. Consequently, the increased carrier density, in conjunction with consistent carrier mobility values, raises electrical conductivity, according to the relation σ = *ne*μ.^1^ A comparable observation was demonstrated by Zhou et al. in Ag-nanoinclusion embedded filled skutterudite, where mobility value slightly increases with the incorporation of Ag-nanoinclusions.^59^ Besides, all nanocomposite films exhibit a rising trend of electrical conductivity with temperature, indicating a nondegenerate semiconductor behavior.

The Seebeck coefficient (*S*) of all thin film samples monotonously increased throughout the entire temperature range except the pristine film, which shows a decrease at 573 K due to intrinsic conduction.^36^ In addition, both pristine and composite films show negative values of *S* representing n-type electronic conduction. In contrast with σ, the *S* values of nanocomposite films decreased with an increasing percentage of the binary telluride phase. The following equation represents the relationship between *S* and carrier density is given by^1^
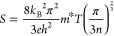
1where m* is the carrier effective mass and *n* is the carrier density. The given relationship reflects the fact that the *S* is inversely proportional to the carrier concentration. As reported earlier, the extrinsic additives alter the carrier concentration but most likely will not affect the effective mass of carriers.^60^ Therefore, the addition of the metallic binary telluride phase only causes change in the effective carrier density of nanocomposite films leading to declined *S* values. Overall, the optimized *S* values can be attained at an optimal ratio between CoTe_2_ and the CGST.

**Figure 4:**
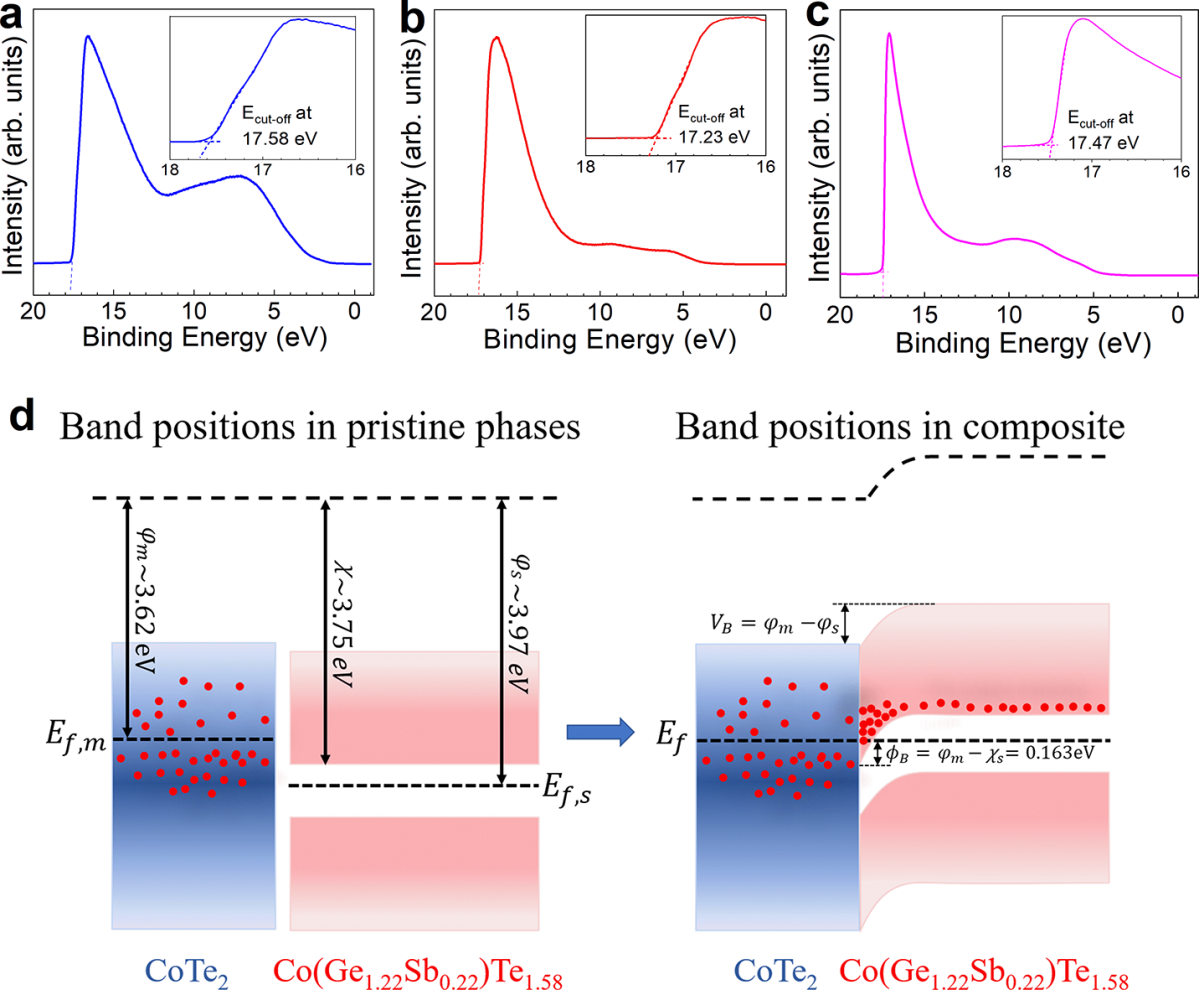
Ultraviolet photoemission spectroscopy (UPS) spectrum of (a) CoTe_2_, (b) pristine Co(Ge_1.22_Sb_0.22_)Te_1.58_, and (c) nanocomposite film *x* = 7; the insets show the close-up region highlighting cutoff energy at the edge. (d) Schematics present the band alignment characteristics between the CoTe_2_ and the CGST matrix with an ohmic contact developed at the interface.

For M–Sc composite, band alignment analysis helps to elucidate the energy level alignment and charge transfer at the interface between the two materials, providing valuable insights into their electronic interactions.^43,44^ A band alignment between the ternary skutterudite and the binary telluride was established by comparing the work functions of both individual materials. The work function (ϕ) of pristine CGST and CoTe_2_ was calculated from the UPS data estimated to be approximately ∼3.97 and ∼3.62 eV, respectively (Figure 4a,b; formula is given in suppoting information). A schematic of the electronic band structure alignment at the interface between the matrix and guest phase is illustrated in Figure 4d. The schematic shows respective Fermi level positions of both phases and conduction and valence band position of the CGST phase, which were taken from the previous report.^16^ For the contact-induced junction, electrons move from a higher Fermi level to a lower Fermi level material. This movement of electrons is driven by the desire to equalize the Fermi levels between the two materials and achieve thermodynamic equilibrium. Under equilibrium, the work function difference vanishes, and the Fermi levels align across the interface. Indeed, the energy offset between the two Fermi levels plays a crucial role in driving the equilibrium charge transfer, as explained by the metal–semiconductor (M–Sc) contact theory.^61^ The lower work function of the metallic CoTe_2_ than the CGST matrix leads to an ohmic contact at the interface, which helps to maintain low interface resistance, and the electrons can flow in either direction without hindrance. It also suggests that electrons have the propensity to flow from the metallic phase to the semiconducting phase and cause band bending at the interface. As a result, this charge transfer leads to an increase in the effective carrier density, enhancing the electrical conductivity values of the composite films. This increased charge density can be corelated experimentally by reduction in work function of nanocomposite film *x* = 7 (3.73 eV) compared to pristine CGST, suggesting that the Fermi level of nanocomposite film shifted upward near conduction band due to excessive electron doping by CoTe_2_, as shown in Figure 4c.

Based on the charge transfer between the CGST and CoTe_2_ phase, the semiconductor bands bend downward and form a small negative potential barrier at the interface, calculated from equation, φ_B_ = ϕ_m_ – χ_SC_, where φ_B_, ϕ_*m*_, and χ_SC_ are barrier height, work function of metal, and electron affinity of semiconducting phase, respectively. This small barrier height at the interface does not cause an adverse effect on the mobility values, thereby leading to less carrier scattering effect and better charge transport, also evident from the measured room temperature carrier density and Hall mobility data. This barrier is also unable to produce hot carrier filtering and plays a minor role in tuning the *S*. These results demonstrate that precise control over the volume fraction and suitable band bending is required to obtain desirable carrier density increment simultaneously with less influence on carrier mobility in related materials.

**Figure 5:**
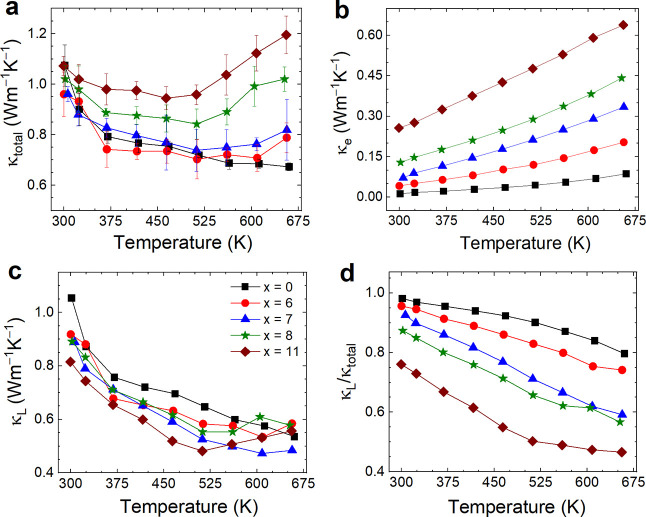
Temperature dependence of (a) total thermal conductivity and (b) the corresponding electronic and (c) lattice components above room temperature. (d) The κ_L_/κ_total_ ratio highlights the lattice contribution.

The total thermal conductivity (κ_total_) values of the pristine and nanocomposite thin films as a function of temperature were measured to study the effect of the thermally conducting CoTe_2_ phase on the pristine CGST phase, are shown in Figure 5a. The κ_total_ values of the pristine film decrease with temperature, whereas for the composite films, it first reduces until 513 K and then starts to increase. However, the κ_total_ was overall higher than pristine for *x* = 7, 8, and 11 films, which embodies larger volume fraction of the metallic telluride phase. This behavior seems obvious due to the domination of the high thermal conducting binary telluride phase (*C*_p_and κ_total_ of CoTe_2_ are shown in Figure S8a).

To clarify the nature of the electronic (κ_e_) and phonon (κ_L_) part of thermal conductivity and evaluate their respective contribution, the κ_total_ was separated in κ_e_ and κ_L_, as displayed in Figure 5b,c. The temperature dependence of κ_e_ values was calculated by the Wiedemann–Franz law, κ_e_ = *L*σ*T*, where *L* is the Lorenz number estimated by the given eq 2,
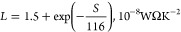
2where *S* is the Seebeck coefficient in μVK^–1^.^62^

Like σ, the κ_e_ also increases with increasing volume fraction of the CoTe_2_ phase, also showing a similar temperature dependency. On the other hand, κ_L_ (calculated using κ_L_ = κ_total_ – κ_e_) of the composite films decreases with the temperature and interestingly shows overall smaller values than the pristine due to increased phonon scattering at the interface. It is also evident from Figure 5d that most of the thermal conduction in the pristine CGST film is governed by lattice vibration. Nearly ∼98% contribution to the κ_total_ for pristine film reduced to ∼76% for *x* = 11, which displays the lowest κ_L_ of 0.48 Wm^–1^ K^–1^ at 513 K. The slope of κ_L_/κ_total_ also changes with temperature, which suggests that the phonon scattering at the interface becomes weaker and the conduction is prominently governed by thermal conducting phase CoTe_2_ at higher temperatures, which also been observed for other composite systems consisting of highly conducting guest phase.^41,63^

**Figure 6:**
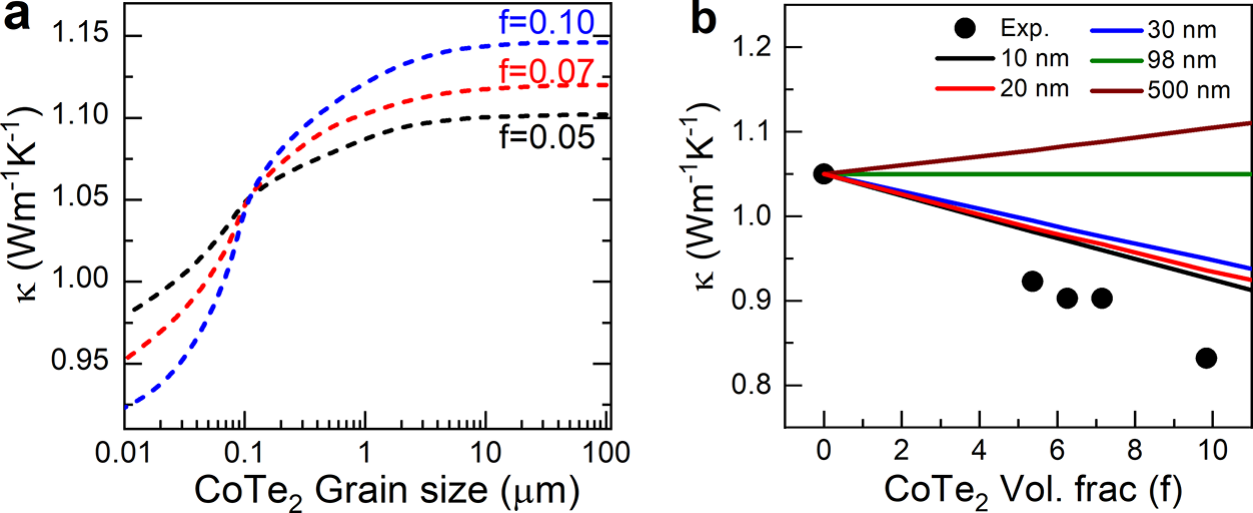
Calculated lattice thermal conductivity for the effect of varied volume faction “*f*” and (a) CoTe_2_ grain size and (b) with experimental data point (solid black circle) at room temperature via Bruggeman’s asymmetrical model considering the interface thermal resistance between the phases.

To understand thermal conduction of a composite system, researchers had utilized the Bruggeman’s asymmetrical model,^64−66^ which considers the interfacial resistance (*R*_int_) between the continuous matrix and the dispersed guest phase. The Bruggeman model allows prediction of total thermal conductivity of composites with varying volume fraction and grain size of the guest phase, as represented in eq 3.
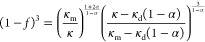
3where *f*, κ_m_, κ_d_, and κ are the volume fraction of dispersed guest phase, lattice thermal conductivity of the matrix, guest phase, and composite, respectively. The parameter  represents the ratio of guest phase grain size and critical radius called Kaptiza radius (*a*_k_ = *R*_int_κ_m_), which holds significant importance in deciding resultant thermal conductivity of composite. When a highly conductive guest phase such as CoTe_2_ introduced into a matrix having relatively lower thermal conductivity values, the effective thermal conductivity of the composite can be reduced below the parent phase value if the particle size of the guest phase is smaller than *a*_k_.^64,65^ This finding led to the prediction of the lattice thermal conductivity of the nanocomposite films in the current study as a function of the grain size with varying volume fraction of CoTe_2_, which is illustrated in Figure 6. It is noted that considering the lattice thermal conductivity of the CGST matrix, the calculated *a*_k_ value for the CGST-CoTe_2_ nanocomposite is determined to be 98 nm at 300 K. This suggests that the phonon thermal conductivity of the composite can be effectively reduced if the particle size of CoTe_2_ is smaller than 98 nm. These results are well corroborated with the experimentally obtained data. The reduction in lattice thermal conductivity of the nanocomposite films can be attributed to the small grain size of CoTe_2_ compared to the Kaptiza radius. This smaller grain size enhances the surface-to-volume ratio, strengthening the effect of *R*_int_ between the phases and leading to increased phonon scattering. However, while the Bruggeman asymmetrical model provides valuable insights, it is important to acknowledge that experimental measurements may exhibit deviations from the model data. The observed 15% deviation in measured thermal conductivity values from the model data suggests the presence of many other factors influencing the thermal behavior of the composite films. Possible factors contributing to this deviation includes microstructural irregularities, variations in particle dispersion, or limitations in the experimental setup and measurement techniques.

**Figure 7:**
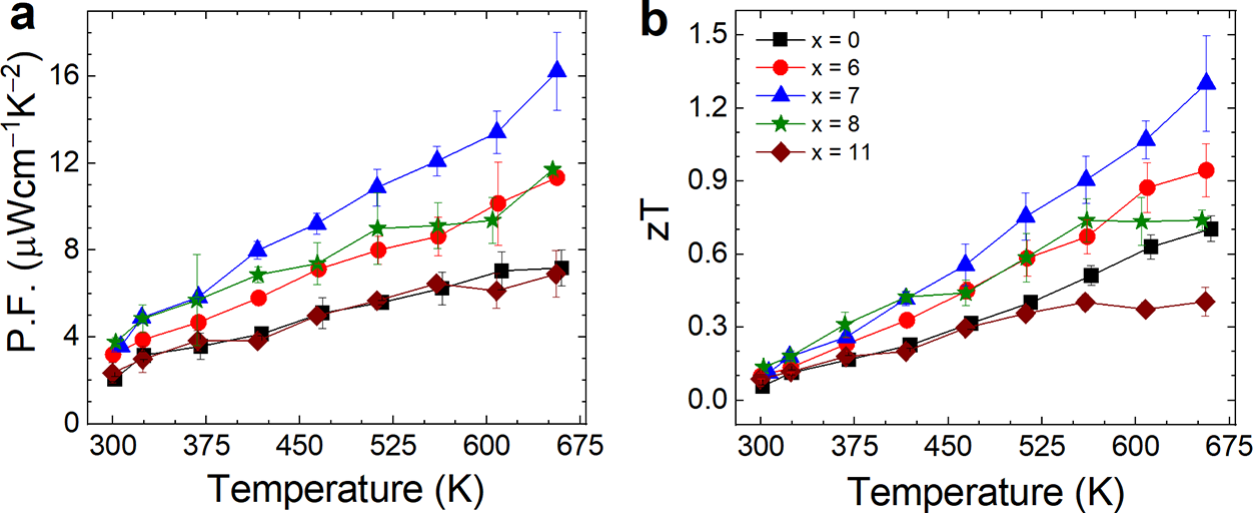
(a) Power factor and (b) zT value of the composite thin films at elevated temperature.

These results reveal that precise control of the band alignment is required to obtain a balanced combination of thermoelectric properties. It should be noted that the suitable alignment and permissible limit of binary telluride phase with ternary skutterudite phase yield a 2-fold increment in power factor of pristine thin film from 7 to 15 μWcm^–1^K^–2^ for *x* = 7 nanocomposite film (Figure 7a). The zT values of the pristine and nanocomposites are also shown in Figure 7b. The zT values of nanocomposite film *x* = 7 significantly improved in the whole temperature range primarily due to an enhanced power factor and reduced lattice thermal conductivity. The best performance had been demonstrated at intermediate levels of extrinsic phase doping, where the mutually interdependent quantities σ, *S*, and κ are perfectly balanced out. Therefore, a maximum zT of 1.30 was achieved at 655 K for the 7 wt. % CoTe_2_-embedded nanocomposite, which is 195% higher than the pristine CGST film.

## Conclusions

We have investigated the thermoelectric properties of the nanocomposite thin films with metal CoTe_2_ and the skutterudite semiconductor Co(Ge_1.22_Sb_0.22_)Te_1.58_ (CGST). Incorporation of metallic CoTe_2_ concurrently optimizes electronic and thermal transport properties of the pristine CGST film, which was deeply investigated in the current study. The electrical conductivity was largely improved resulting in a 2-times increment in power factor compared to the pristine CGST film. This enhancement is attributed to the synergetic effect of charge transfer and moderate scattering of conduction electrons at the interface facilitated by the small potential barrier and ohmic band alignment between CGST and CoTe_2_. Phonon scattering, on the other hand, grows with the fraction of CoTe_2_ and thereby the volume of the interface, leading to an effectively reduced lattice thermal conductivity. The maximum zT of 1.30 exists in the 7 wt. %-CoTe_2_ film at 655 K. Our work highlights how important a precise control over the volume fraction of the secondary phase and electronic band alignment is to achieve a metal–semiconductor nanocomposite for efficient thermoelectric conversion.

## Supporting Information Available

Atomically resolved high-angle annular dark-field (HAADF) images of “*x* = 7” nanocomposite; Rietveld refinement XRD patterns of nanocomposite films for *x* = 0, 7, and 11; refinement parameters for CGST(1–*x*)-CoTe_2_(*x*) nanocomposite films; EPMA composition of CGST(1–*x*)-CoTe_2_(*x*) nanocomposite films; Raman spectra of CGST(1–*x*)-CoTe_2_(*x*) nanocomposite films; extended 2θ range (5–40°) and enlarged 2θ window XRD patterns of CGST(1–*x*)-CoTe_2_(*x*) nanocomposite films; XPS core-level of Co 2p and Te 3d of nanocomposite films for *x* = 0 and 7; cross-section SEM images of nanocomposite films; cross-section TEM-EDS maps of “*x* = 7” nanocomposite; thermal and electronic properties of hot-presses CoTe_2_; surface topology and corresponding current distribution map for *x* = 7 and 11 nanocomposite films; cyclic reproducibility of electronic properties for 3 different batches of “*x* = 7” nanocomposite thin film; work function calculation from UPS measurement; thin film thermal conductivity measurement method (TDTR) description; volume fraction calculation formula for Bruggeman model; temperature-dependent specific heat and density values of CGST(1–*x*)- CoTe_2_(*x*) nanocomposite films, calculated using rule of mixture formula (PDF)
