# Supplementary material for: Boosting Thermoelectric Performance in Nanocrystalline Ternary Skutterudite Thin Films through Metallic CoTe2 Integration
Source: ACS Appl Mater Interfaces. 2024 Mar 15;16(12):14770–80. doi: 10.1021/acsami.3c17695 (PMC10982935; doi:10.1021/acsami.3c17695)
Supplement: Supplementary file 1 — am3c17695_si_001.pdf [file am3c17695_si_001.pdf]

Boosting Thermoelectric Performance in Nanocrystalline Ternary Skutterudite Thin Films through Metallic $CoTe_2$ Integration

Bhawna Jarwal [1, 2, 3, 4], Suman Abbas [1, 3, 4, 5], Ta-Lei Chou [4], Suneesh M. Vailyaveettil [3], Ashutosh Kumar [6], Shaham Quadir [4, 7], Thi-Thong Ho [3], Deniz P. Wong [8], Li-Chyong Chen [4, 9, 10], Kuei-Hsien Chen [3, 4]**

[1]Molecular Science and Technology Program, Taiwan International Graduate Program, Academia Sinica, Taipei 10617, Taiwan.

[2]Department of Molecular Science and Technology, National Taiwan University, Taipei 10617, Taiwan.

[3]Institute of Atomic and Molecular Sciences, Academia Sinica, Taipei 10617, Taiwan.

[4]Center for Condensed Matter Sciences, National Taiwan University, Taipei 10617, Taiwan.

[5]Department of Physics, National Central University, Taoyuan 32001, Taiwan.

[6]Department of Materials Science and Metallurgical Engineering, Indian Institute of Technology Bhilai, Durg-491001, Chhattisgarh, India.

[7]Materials Science Center, National Renewable Energy Laboratory (NREL), Golden CO, 80401, USA.

[8]Helmholtz-Zentrum Berlin für Materialien und Energie, Hahn-Meitner-Platz 1, D-14109 Berlin, Germany.

[9]Department of Physics, National Taiwan University, Taipei, 10617, Taiwan.

[10]Center of Atomic Initiative for New Materials, National Taiwan University, Taipei, 10617, Taiwan.

* The corresponding author's e-mail: chenkh@pub.iams.sinica.edu.tw; chenlc@ntu.edu.tw

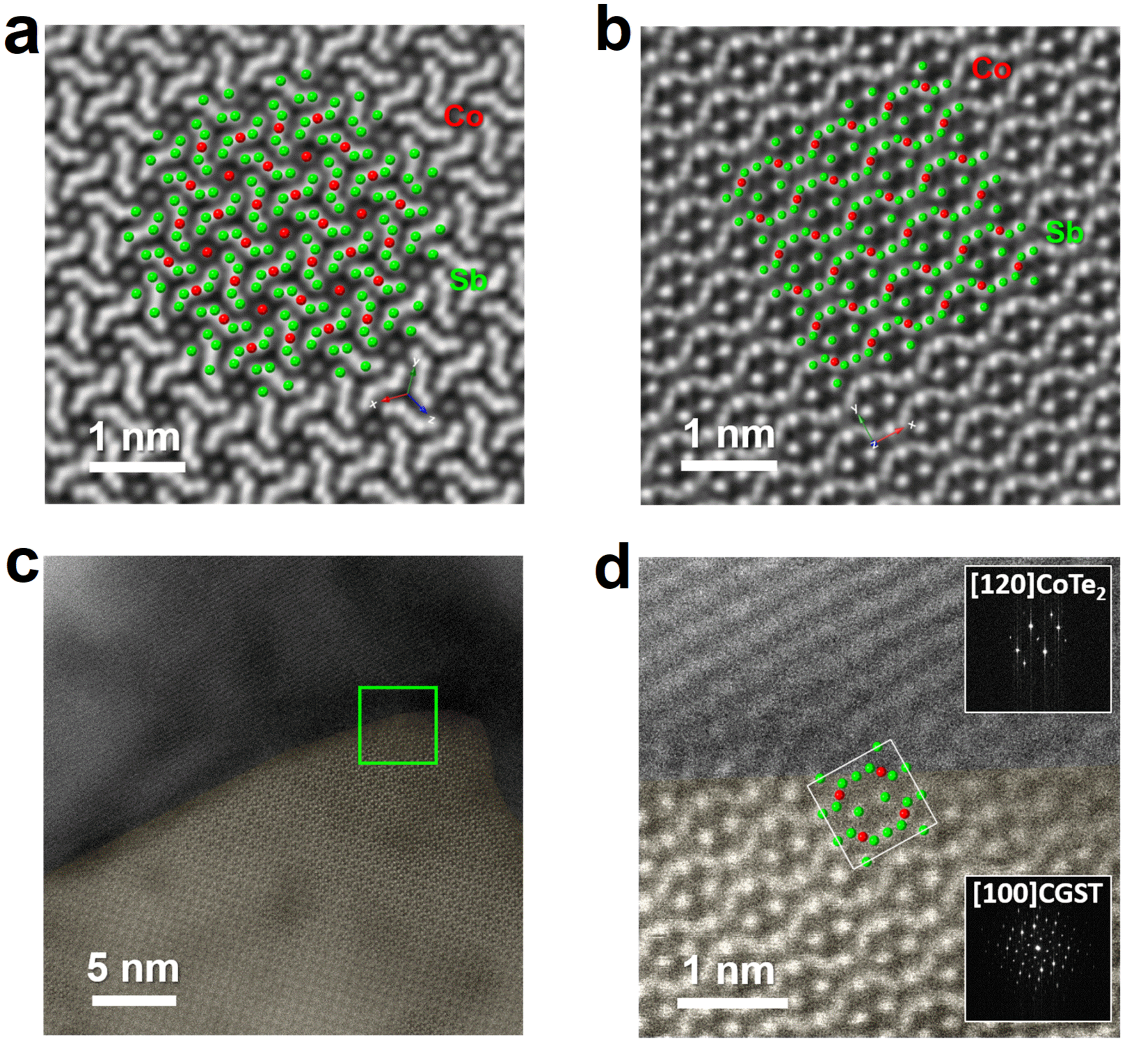

Figure S1. The atomically resolved high-angle annular dark-field (HAADF) images for CGST along different zone axes. On top of them, the atomic structure from skutterudite-type CoSb3 (space group Im-3, No.204) unambiguously points out that the dopant Sb turns rhombohedral Co-Ge-Te (space group R-3H, No.148) phase into cubic and the view directions of (a) and (b) are [111] and [100], respectively, The high-angle annular dark-field image spotting the interface between a CGST grain, as highlighted in yellow, and neighboring $CoTe_2$ (c). In the blowup (d),

the Fast-Fourier Transforms (FFTs) from the upper and lower half indicate the orientations are $[120]_{CoTe2}$ and $[100]_{CGST}$, respectively.

XRD measurement and analysis details:

The XRD patterns of thin films were collected at the Taiwan Synchrotron Facility NSRRC by photon energy of 20 KeV (0.61992 Å), therefore, the current 2θ values are obtained at a high energy photon source. The respective 2θ values from Figure 1a, fall in the range of 12-65° in CuKα energy. Additionally, we are providing here a large 2θ spectrum from 5-40° (CuKα range: 12-116°) in Figure S3a.

The refinement fitting process was conducted on TOPAS v5 software using the Rhombohedral CIF file of the ternary skutterudite phase. In the pristine structure, this phase crystallizes in $R\overline{3}$ symmetry. However, as we reported in our previous work (Inorganic Chemistry 2022, 61 (10), 4442-4452), the introduction of Sb doping restores the $R\overline{3}$ symmetry to $Im\overline{3}$ symmetry. The unavailability of an Im3 CIF file led us to utilize the R3 CIF file for the refinement process.

In the current XRD patterns of the thin films, an absence of Rhombohedral structure reflections at specific 2θ peaks (12.247° and 13.543°) was observed. This observation strongly indicates that the thin films have a cubic structure. Therefore, additional peaks observed in the difference pattern of

the fitting arise from the absence of these R3 symmetry reflections in the experimental data. Given our primary objective of estimating the weight fraction of $CoTe_2$, we decided to use the R3 CIF file for fitting our experimental XRD patterns. This approach, while unconventional, was carefully considered and executed. We ensured the goodness of the fitting by achieving R_{wp} values below 7%.

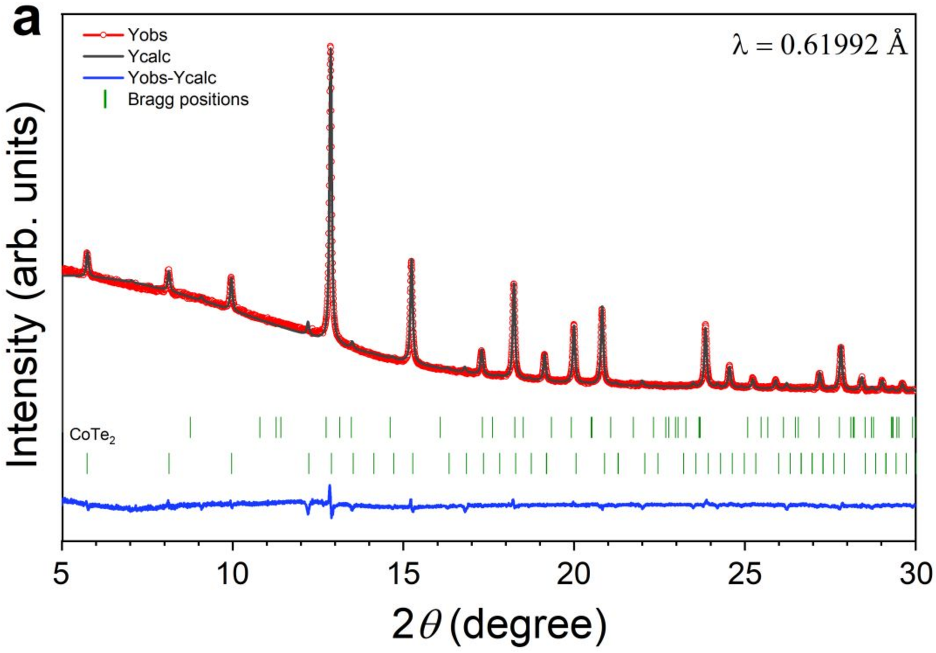

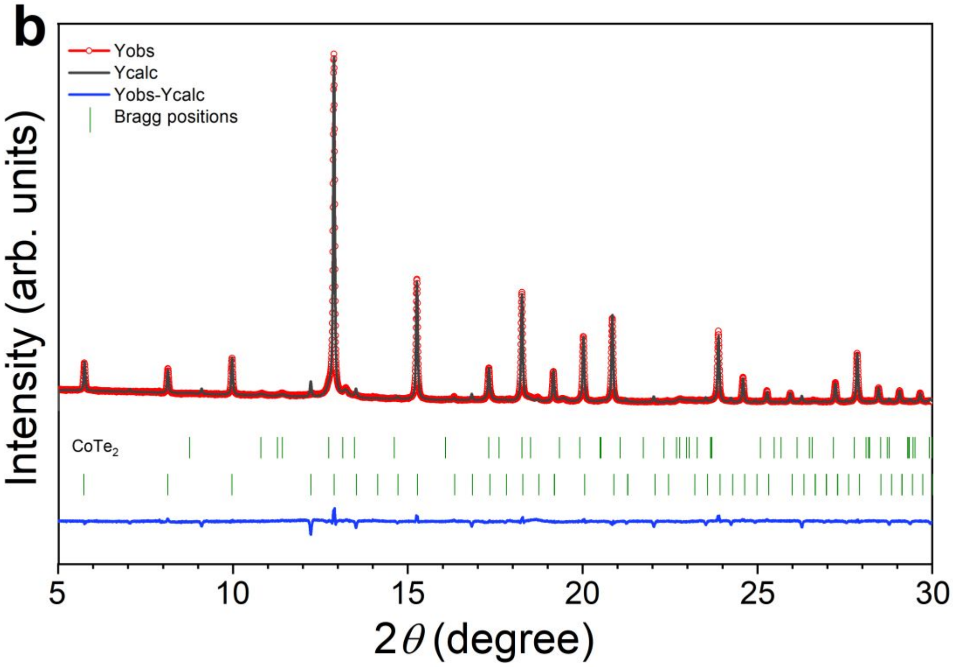

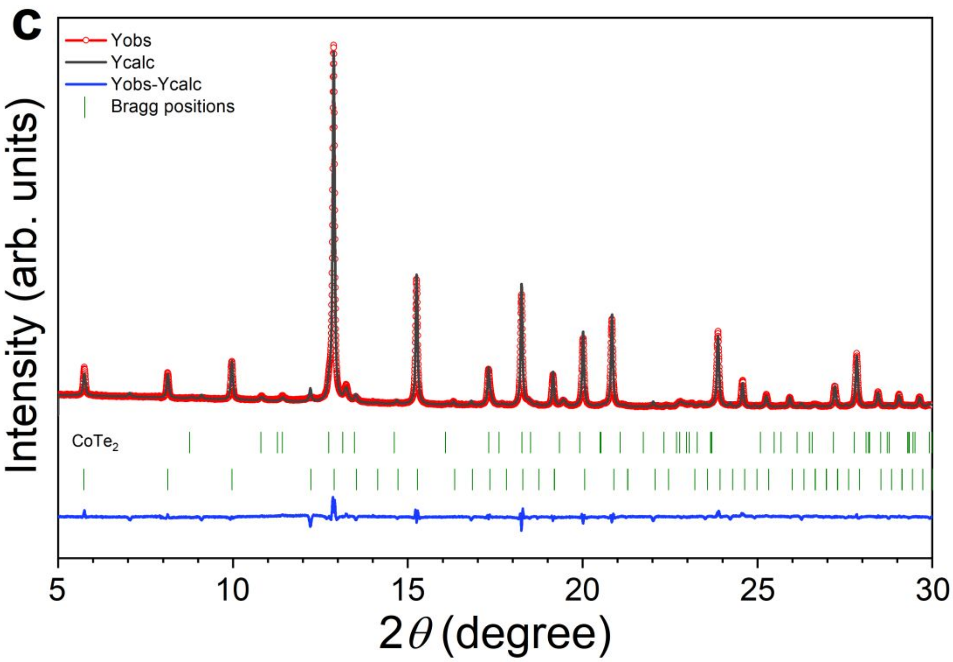

Figure S2. Rietveld refinement XRD patterns of nanocomposite films for x= 0, 7 and 11 (a)–(c), respectively.

Table S1. Refinement parameters R_{wp}, Lattice parameters a, c and volume of unit cell for (1-x) CGST with x $CoTe_2$ composite sample calculated from the Rietveld refinement of the XRD patterns.

CGST (1-x)- $CoTe_2$ (x)		a (Å)	*b* (Å)	*c* (Å)	V(Å^3)	R_{wp}	Phase %
x = 0	CGST	12.369(3)	-	15.146(3)	2007.089	2.080	100
	$CoTe_2$	-	-	-	-		0
x = 6	CGST	12.349(4)	-	15.131(5)	1998.555	6.684	93.81
	$CoTe_2$	5.276(5)	6.258(5)	3.864(4)	127.605		6.19
x = 7	CGST	12.357(2)	-	15.137(4)	2001.788	5.542	92.73
	$CoTe_2$	5.262(2)	6.254(5)	3.855(3)	126.910		7.27
x = 8	CGST	12.355(4)	-	15.138(4)	2001.471	6.389	91.90
	$CoTe_2$	5.265(4)	6.256(3)	3.865(3)	127.323		8.10
x = 11	CGST	12.362(4)	-	15.124(2)	2001.786	5.412	88.77
	$CoTe_2$	5.265(3)	6.254(2)	3.862(5)	127.195		11.23

Table S2. EPMA composition of nanocomposite films for x= 0, 6, 7, 8, and 11.

Atomic %	Co	Ge	Sb	Te
x = 0	25.39 ± 0.18	30.03 ± 0.04	5.63 ± 0.01	38.95 ± 0.15
x = 6	25.18 ± 0.47	25.25 ± 0.36	5.45 ± 0.06	44.11 ± 0.16
x = 7	25.67 ± 0.35	23.69 ± 0.17	5.03 ± 0.06	45.61 ± 0.56
x = 8	25.28 ± 0.04	23.01 ± 0.03	4.87 ± 0.05	46.85 ± 0.06
x = 11	25.11 ± 0.49	23.14 ± 0.51	4.60 ± 0.12	47.15 ± 0.15

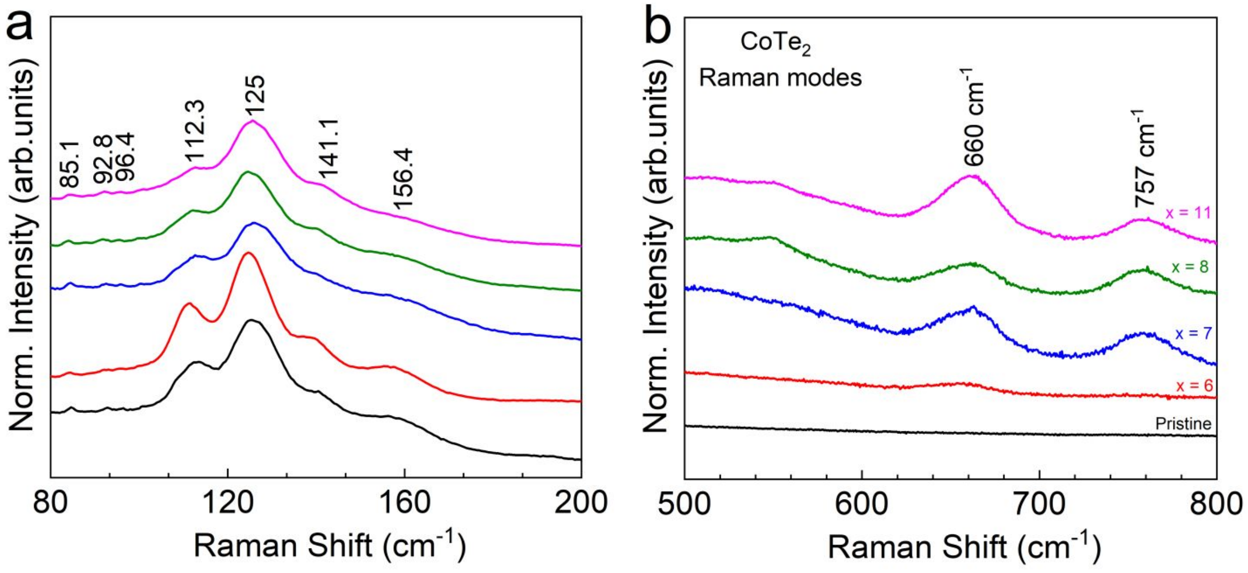

Figure S3. Raman spectra of nanocomposite films for x= 0, 6, 7, 8, and 11, showing Raman modes of $Co(Ge_{1.22}Sb_{0.22})Te_{1.58}$ (a) and $CoTe_2$ (b).

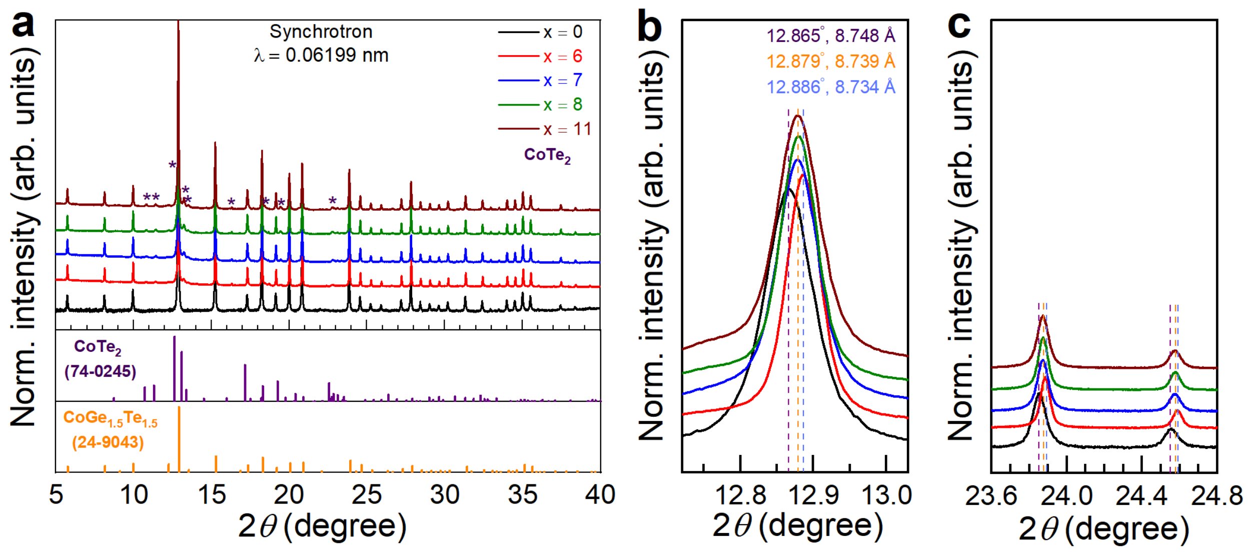

Figure S4: XRD patterns of CGST with x wt.% of $CoTe_2$ (x= 0, 6,7,8, and 11) composite in the 2θ range of 5-40° (CuKα range: 12-116°) (a) and enlarged 2θ window at CGST diffraction peaks in different range (b) and (c).

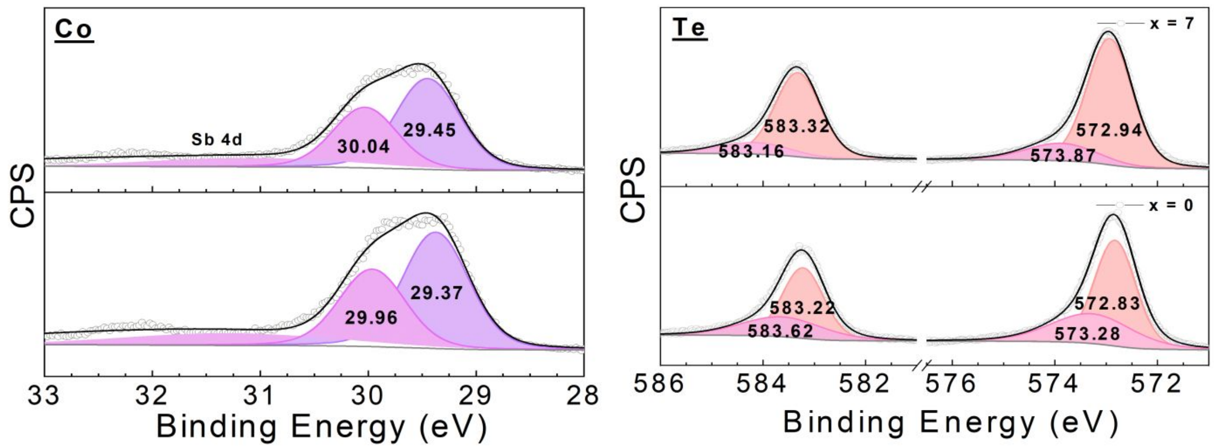

Figure S5. XPS core-level of Co 2p (a) and Te 3d (b) of nanocomposite films for x =0 & 7.

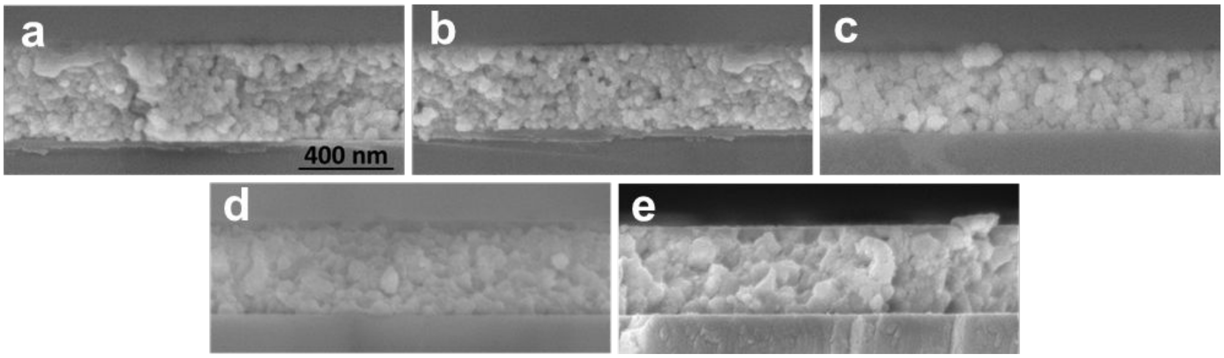

Figure S6. Cross-section SEM images of nanocomposite films for x= 0, 6, 7, 8, and 11 (a)-(e).

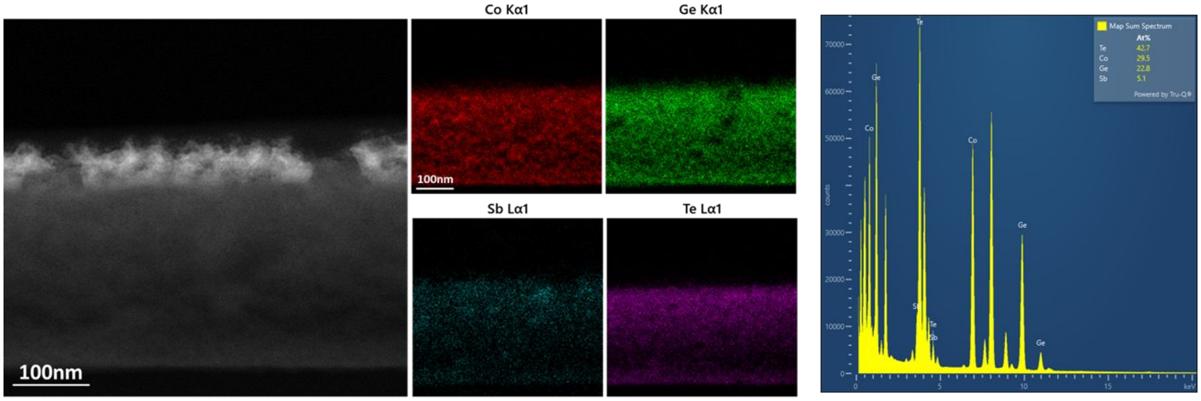

Figure S7. Cross-section TEM-Eds elemental maps of x=7 nanocomposite thin film.

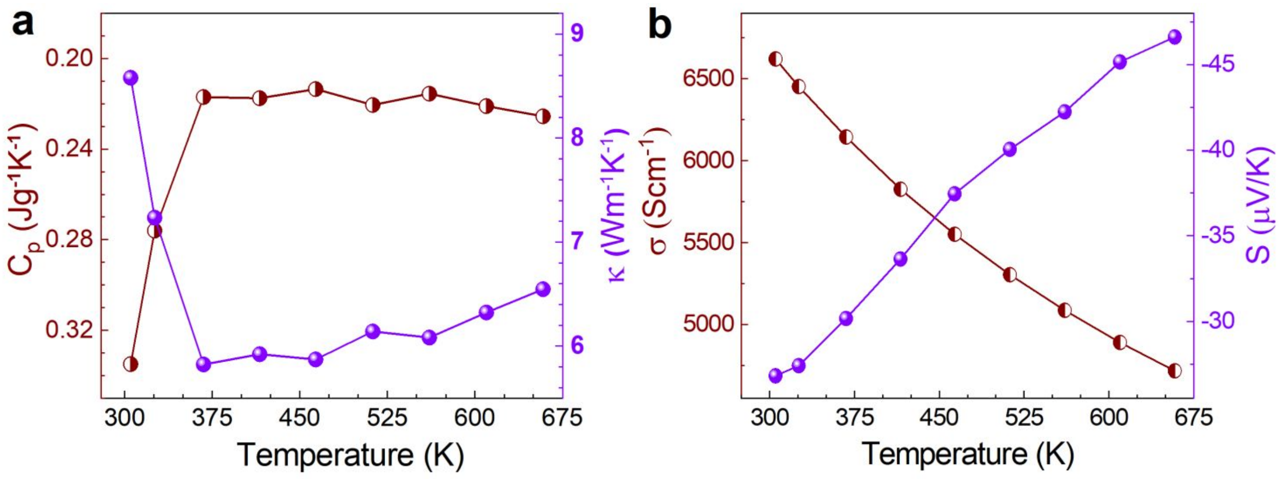

Figure S8. Thermal (a) and Electrical (b) transport properties of hot-pressed $CoTe_2$.

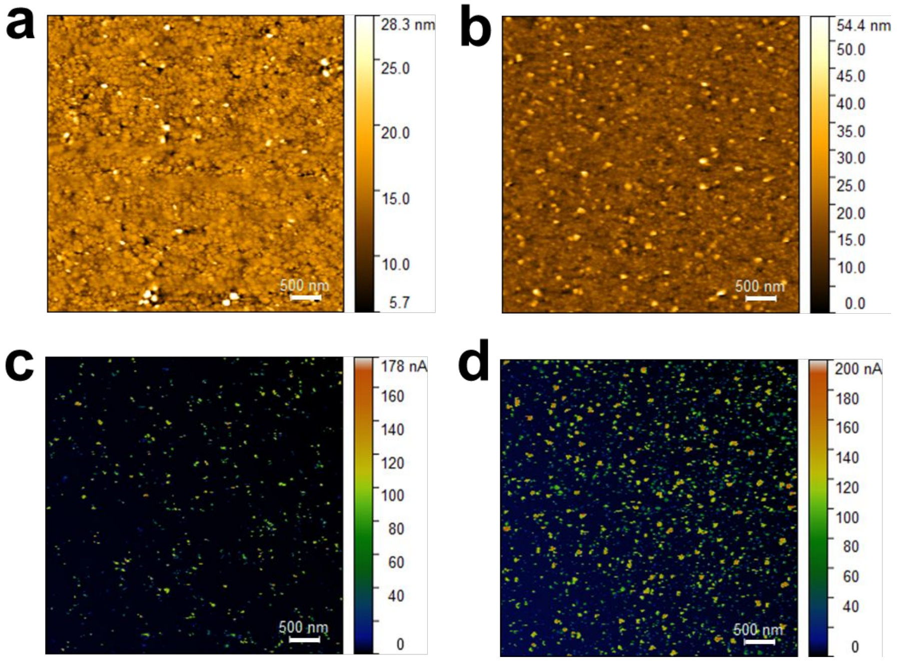

Figure S9. Surface topology (a) and (b) and the corresponding current distribution map (c) and (d) for nanocomposite films x= 7 and 11.

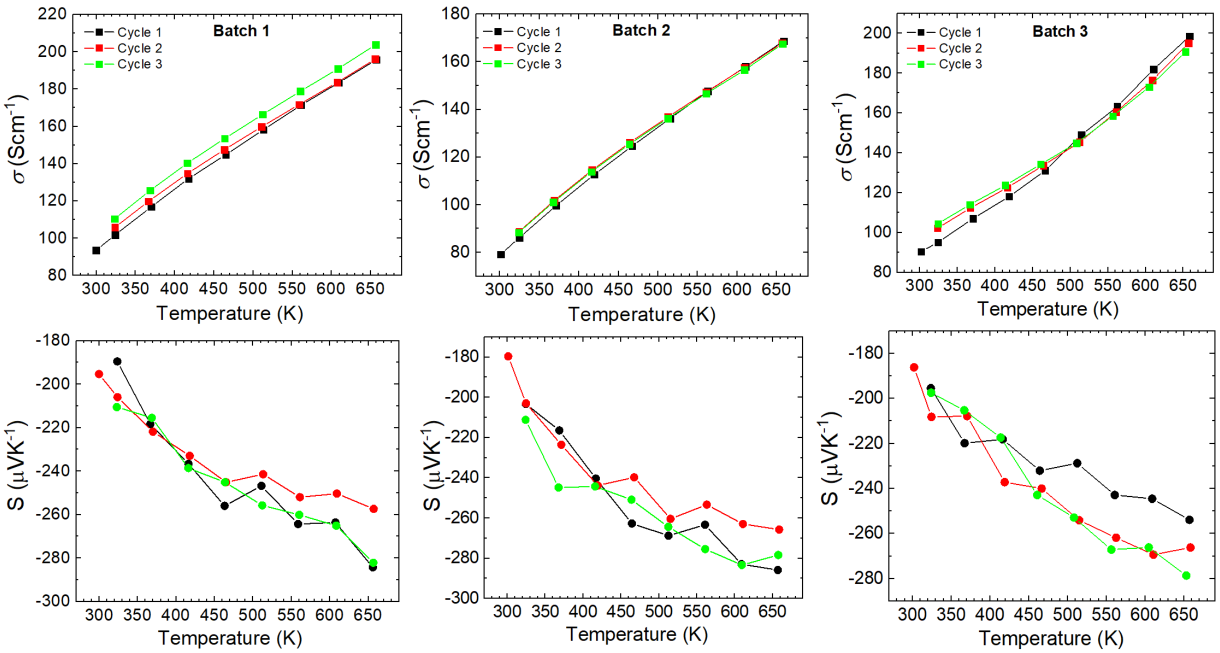

Figure S10. Temperature dependence of electric conductivity and Seebeck coefficient for 3 different batches of x=7 nanocomposite thin film.

Band alignment analysis:

The work function (ϕ) of respective phases were calculated from the measured UPS data using the given equation[1,2] –

$$\phi = h\nu - E_{cut-off}$$

where ($h\nu$ = 21.2 eV) is the He-light source photon energy, and $E_{cut-off}$ is the binding energy of cut-off measured by UPS measurement represented in Figures 4a and b.

TDTR measurement:

In our study, we employed the Time-domain Thermo-reflectance (TDTR) method to measure the thermal conductivity of our thin films. The TDTR technique is based on a pump-probe approach. It involves using a short laser pulse, referred to as the 'pump', to heat the surface of the known transducer layer on top of the subjected thin film sample. Subsequently, a delayed laser pulse, known as the 'probe', is used to measure the change in reflectance caused by the temperature change on the sample surface. By analyzing the temporal evolution of the surface temperature, we can derive critical information about the thermal properties of the materials under investigation.

A key aspect of accurately measuring the thermal conductivity of the thin film, while effectively excluding the influence of the substrate, lies in the use of a sophisticated three-layer heat diffusion model. The experimental data obtained from TDTR is fitted using the provided model. This model comprehensively accounts for the thermal contributions from both the thin film and the substrate. By incorporating known properties of the substrate, such as its thickness and thermal conductivity, into the model, we can isolate and accurately determine the thermal conductivity of the thin film alone. This approach ensures that the measured thermal properties are representative of the thin

film itself, effectively minimizing the impact of the substrate on our results. Further model details can be found in the following research article {David. G Cahill Rev.Sci. Instrum., 75, 12, 2004}.

In the current study, a 200 nm thick Au layer followed by 5 nm Cr was deposited on nanocomposite thin film samples to use as a transducer layer. The measurement quality and reliability are ensured by an average of 5 data collection points at each temperature of the subjected sample.

For Bruggeman model:

The volume fraction was determined using the formula:

$$\frac{\text{Weight fraction}_{\text{CoTe2}}}{\text{density}_{\text{CoTe2}}} \text{ x density}_{\text{CGST}}$$

Table S3. Temperature-dependent specific heat and density values of pristine CGST, $CoTe_2$, and of x= 0, 6, 7, 8, and 11 nanocomposite films, calculated by rule of mixture formula.

Temp. (K) / Cp (J/Kg.K)	$Co(Ge_{1.22}Sb_{0.22})Te_{1.58}$	$CoTe_2$	x = 6	x = 7	x = 8	x = 11
303	276	335	279.54	280.13	280.72	282.49
323	292	276	291.04	290.88	290.72	290.24
373	307	217	301.60	300.70	299.80	297.10
423	281	217.5	277.19	276.56	275.92	274.02
473	279	213.5	275.07	274.42	273.76	271.80
523	276	220.5	272.67	272.12	271.56	269.90
573	289	215.5	284.59	283.86	283.12	280.92
623	291	221	286.80	286.10	285.40	283.30
673	293	225.5	288.95	288.28	287.60	285.58
Density @RT						
g/cm^3	7.03	7.93	7.084	7.093	7.102	7.129

References:

1. Moyzhes, B.; Nemchinsky, V. Thermoelectric figure of merit of metal–semiconductor barrier structure based on energy relaxation length. Applied physics letters 1998, 73 (13), 1895-1897.
2. Liu, Y.; Cadavid, D.; Ibáñez, M.; Ortega, S.; Martí-Sánchez, S.; Dobrozhan, O.; Kovalenko, M. V.; Arbiol, J.; Cabot, A. Thermoelectric properties of semiconductor-metal composites produced by particle blending. Apl Materials 2016, 4 (10), 104813-104817.
